# Complex-Valued Wavelet Spectrum Analysis of Respiratory Conditions and Its Feasibility in the Detection of Low-Functional Respiration

**DOI:** 10.3390/healthcare9080981

**Published:** 2021-08-02

**Authors:** Yoshikazu Nakajima, Takaaki Sugino, Masashi Kobayashi, Yasuhiro Nakashima, Yohei Wada, Yasuro Okumiya, Koji Yataka, Katsunori Suzuki, Toshihiro Kawase, Shinya Onogi, Kenichi Okubo

**Affiliations:** 1 Department of Biomedical Information, Tokyo Medical and Dental University, Tokyo 101-0062, Japan; sugino.bmi@tmd.ac.jp (T.S.); kawase.bmi@tmd.ac.jp (T.K.); onogi.bmi@tmd.ac.jp (S.O.); 2 Department of Thoracic Surgery, Tokyo Medical and Dental University, Tokyo 113-8510, Japan; mkobthsr@tmd.ac.jp (M.K.); midysland@gmail.com (Y.N.); okubo.thsr@tmd.ac.jp (K.O.); 3 Department of Thoracic Surgery, Kurashiki Central Hospital, Okayama 710-8602, Japan; 4 Research & Development Division, Yamaha Corporation, Shizuoka 430-0904, Japan; yohei.wada@music.yamaha.com (Y.W.); yasuro.okumiya@music.yamaha.com (Y.O.); koji.yataka@music.yamaha.com (K.Y.); katsunori.suzuki@music.yamaha.com (K.S.)

**Keywords:** respiration activity monitoring, respiratory condition, wavelet transform, correlation evaluation

## Abstract

Respiratory monitoring is a significant issue to reduce patient risks and medical staff labor in postoperative care and epidemic infection, particularly after the COVID-19 pandemic. Oximetry is widely used for respiration monitoring in the clinic, but it sometimes fails to capture a low-functional respiratory condition even though a patient has breathing difficulty. Another approach is breathing-sound monitoring, but this is unstable due to the indirect measurement of lung volume. Kobayashi in our team is developing a sensor measuring temporal changes in lung volume with a displacement sensor attached across the sixth and eighth ribs. For processing these respiratory signals, we propose the combination of complex-valued wavelet transform and the correlation among spectrum sequences. We present the processing results and discuss its feasibility to detect a low-functional condition in respiration. The result for detecting low-functional respiration showed good performance with a sensitivity of 0.88 and specificity of 0.88 to 1 in its receiver operating characteristic (ROC) curve.

## 1. Introduction

The monitoring of respiratory conditions is a common concern in patient care in postoperative treatments and epidemic infections such as COVID-19. After surgery, patients can be in an unstable condition, and if their condition worsens, treatment should start within minutes. Frequent checks for a patient’s condition are important to start speedy treatment. Another case that requires respiratory monitoring is COVID-19, which poses a threat to the world and could cause widespread infections. This can be submerged and incubate for up to a couple of weeks, but possibly makes the respiration activity of affected people severe in a couple of hours. For speedy medical care, medical staff are burdened with frequently checking patients’ respiratory conditions, such as every hour for a long period of around two weeks. The automatic monitoring of respiratory activation is required to reduce patient risks and the difficulty of medical staff labor. Oximetry is widely used in automatic respiratory monitoring, but it does not directly capture respiratory motion. There were some cases in which oximetry did not detect low-functional respiratory condition even though the patient declared difficulty in breathing. This could be observed in having introduced breathing assistance. Breathing-sound monitoring was reported to detect asthma attacks [1,2]. The combination of breathing sound and chest movement estimated the breathing period but did not evaluate respiratory activity. Temperature-based sensors were also implemented to monitor respiratory rate but were still an indirect measurement of breathing volume [3]. Other approaches addressed this with wearable or ultrasound-based surface-measuring sensors but could have problems f looseness or instability [4,5,6,7]. To directly measure lung volume, although this represents one dimension of the volume, Kobayashi et al. detected lung volume over time by measuring changes in the intercostal distance by using a displacement sensor attached across the sixth to eighth ribs [8,9]. They applied Hilbert and Fourier transforms to the measured signals and confirmed a signal-pattern difference between normal and low-functional lung phases. Their results showed some feasibility to be applied to monitor respiratory activation, but some improvements were needed on sensitivity and stability to apply to the clinic. Regarding previous approaches to describe wavy signals in medicine, frequency-domain transforms are promising techniques due to their high temporal resolution and stability against phase shift. A conventional Fourier transform is commonly used, but Fedzai [10] introduced short-time Fourier transform for brain EEG analysis to improve the capability of time-varying description for wavy signals. Yamamoto [11] introduced short-time Fourier transform as preprocessing with a long-short term memory (LSTM) neural network for counting the heartbeat rate. Compared with both conventional and short-time Fourier transforms, wavelet transform can provide a higher temporal resolution. Warrick applied wavelet transform to detect sleep disorders with sleep-arousal-in-polysomnographic (PSG) signals [12]. For highlighting another aspect of the wavelet transform, Bao [13] and Qiu [14] employed wavelet transform to eliminate high-frequency noise and long-term drift for economic forecasting. Wang used wavelet transform to reduce noise in motor signals [15]. Yildirim et al. used the combination of wavelet transform and LSTM for ECG brain signal classification [16]. Regarding wavelet transformation options, a complex-valued feature compensates for the phase shift for each wavelet frequency component. Therefore, in this study, we evaluate respiratory signals with complex-based wavelet transform to clarify the analytical capability of the complex-valued wavelet transform and show a criterion with the frequency domain to detect low-functional conditions in respiration.

## 2. Method of Respiration Phase Classification

### 2.1. Acquirement of Respiration Signals

**Figure 1:**
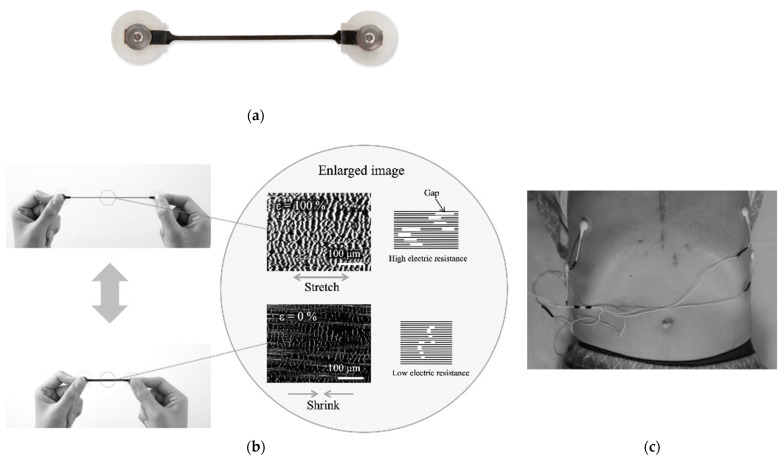
Displacement sensor developed by Yamaha Co.: (**a**) appearance; (**b**) functional principle; (**c**) scene of attaching a sensor to a patient.

A stretch sensor, as shown in Figure 1, was used to capture patient respiration. This was developed at Yamaha Corporation and Shizuoka University [17,18]. It is composed of multiwall carbon nanotubes, shown as the black part in Figure 1a, and can stretch itself to up to 200% of its length, as shown in Figure 1b. The frosted white parts, made of plastic, are fixed onto a patient’s skin. The metal parts were connected to the electric cables to measure the stretch part’s electrical resistance. The sensor linearly reported the displacement between its two edges as resistance variation with very small hysteresis. One edge was placed with a biocompatible adhesion sheet onto the skin at the 6th rib and the other was at the 8th rib, as shown in Figure 1c. The sensor showed a resistance difference for following the 6th to 8th intercostal distance which reveals a correlation with respiration phases. We collected respiration signals with the 6th to 8th rib distance under the supposition that inter-rib distance could be linear with respect to lung volumes. Data samplings for respiratory signals were taken at 10 Hz frequency.

### 2.2. Complex-Valued Wavelet Transform

**Figure 2:**
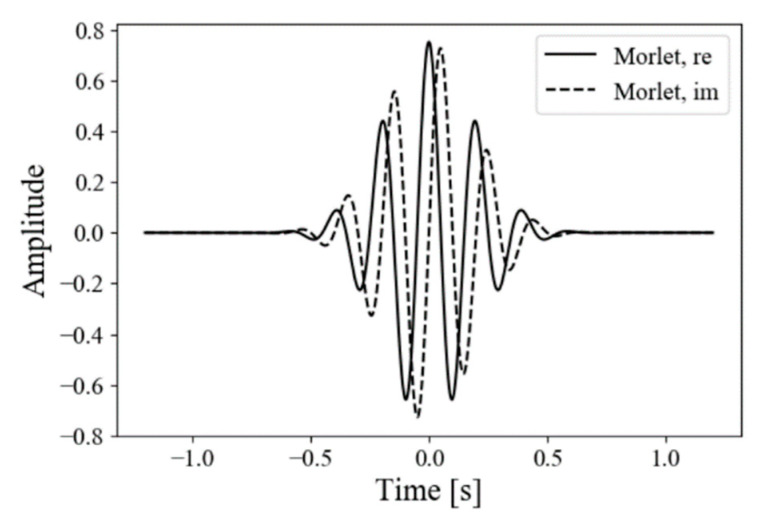
Example of Morlet wavelet kernel: line and dashed line show real and imaginary components, respectively.

Complex-valued wavelet transform (CWT) [18] processes signals of intercostal distance. It converts the original signals to time-varying representation in the frequency domain. The mother wavelet was a Morlet kernel function as shown in Figure 2, which consisted of a plane wave modulated with a Gaussian:

(1) $$\psi_{\mathrm{Morlet}}\left(\eta ;\omega_{0}\right)=e^{\frac{-\eta^{2}}{2}}e^{i\omega_{0}\eta }$$

where *η* is the past phase for the origin of the kernel function, and $\omega_{0}$ is the constant specifying wavelet frequency. Since Napier’s constant imaginarily powered means a complex trigonometric wave function,$\;\psi_{\mathrm{Morlet}}\left(\eta ;\omega_{0}\right)$ can be decomposed as

(2) ψMorlet(η;ω0)=Re(ψMorlet(η;ω0))+i Im(ψMorlet(η;ω0))

where

(3) Re(ψMorlet(η;ω0))=e−η22cos(ω0η)

is for the real components, and

(4) Im(ψMorlet(η;ω0))=e−η22sin(ω0η)

is for the imaginary components.

Considering wavelet scale s and time-shift phase t, η is given as $\frac{\zeta -t_{0}}{s}$ with pseudotime ζ for wavelet functions. Thus, the wavelet function is given as

(5) $$\psi \left(\zeta ;s,t,\omega_{0}\right)=\frac{1}{\sqrt{s}}\psi_{\mathrm{Morlet}}\left(\frac{\zeta -t}{s};\omega_{0}\right)$$

where s is given from frequency f with sampling period Δ and pseudofrequency $f_{\mathrm{pseudo}}$ corresponding to the scale s as

(6) $$s=\frac{f}{f_{\mathrm{pseudo}}\;\Delta }$$

Then, the dot product for time-domain signal x(t) with the wavelet function gives a complex vector of the frequency domain spectrum as

(7) $$W\left(f;t\right)=\langle x\left(\zeta \right)|\psi \left(\zeta ;s,t\right)\rangle =\int_{\mathbb{R}}^{}x\left(\zeta \right)\psi \left(\zeta ;s,t\right)d\zeta$$

where t is the time for signal function x(t), and 〈 ·|· 〉 is a dot product. It is input to the wavelet function as the time shift. W(f) is a complex-valued vector that contains complex frequency components.

### 2.3. Detection of Low-Functional Respiration

**Figure 3:**
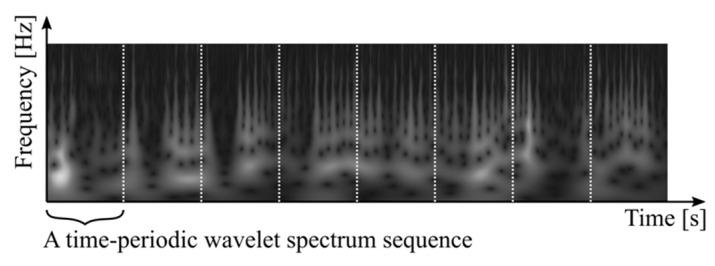
Generation of periodic data segments of respiratory wavelet spectrum sequence.

Respiratory signals might repetitively vary with arbitrary time duration. A normal respiratory signal repeats around every 4–5 s [19]. A repetitive signal can also appear around every 40–50 s [9] or at a longer interval, particularly in low-functional respiration according to empirical knowledge in the clinic. This leads to a signal-processing strategy that is the correlation of time-periodic spectral series being able to distinguish normal and low-functional respirations. Thus, our implementation began to segment the spectral time sequence to periodic datasets with arbitrary time duration, as shown in Figure 3. Let $t_{d}$ be an arbitrary time duration of periodic data $S_{W}\left(t;\;t_{d}\right)$; it can be denoted as

(8) $$S_{W}\left(t;\;t_{d}\right)=\left\{W\left(f;t+\Delta t\right)\;|\;f>0,\;0\leq \Delta t\leq t_{d},\;f\in \mathbb{R},\;\Delta t\in \mathbb{R}\right\},$$

where Δt is the past time from the time when the periodic data start. Then, the periodic datasets are averaged with respect to discrete time with interval $t_{d}$, which equals the time duration of the data period, and derives averaged periodic data $\bar{S_{W}}$ as

(9) $$\bar{S_{W}}\left(t_{d}\right)=\frac{1}{n_{f}}\overset{}{\underset{t}{\sum }}S_{W}\left(t;\;t_{d}\right),$$

where $n_{f}$ is the number of periodic data segmented in the entire time sequence of the respiratory wavelet spectrum. $S_{W}$ is then evaluated with correlation criteria to detect a low-functional period from normal respiration. The normalized correlation coefficient (NCC) gives the signal correspondence of $S_{W}\left(t;\;t_{d}\right)$ and its average as

(10) $$NCC\left(S_{W}\left(t;\;t_{d}\right),\bar{S_{W}}\left(t_{d}\right)\right)=\langle \tilde{S_{W}}\left(t;\;t_{d}\right)|\tilde{\bar{S_{W}}}\left(t_{d}\right)\rangle$$

where $\tilde{\cdot }$ is a normalizing operation for both frequency and time. 〈 ·|· 〉 means the dot product of vector expressions of two functions with respect to both frequency and time. Since the respiration signal is stable in normal respiration and varies in low function, $NCC\left(S_{W}\left(t;\;t_{d}\right),\bar{S_{W}}\left(t_{d}\right)\right)$ possibly detected a low-functional period. The criteria showed high values in stable respiratory conditions, such as normal respiration, and low values in time-varying respiratory conditions, such as low-functional respiration.

## 3. Experiments

### 3.1. Difference Analysis between Normal and Low-Functional Respiration Periods with Wavelet Transform

The respiration data of 19 patients, who were 61 to 80 years old, were evaluated. They were classified by a respiratory specialist or medical doctor with a criterion on featured wave shapes written in [9]. Data conditions are shown in Table 1. They comprise 206 periods for normal respiration and 92 periods for low-functional respiration. The time durations of low-functional respiration periods tended to be shorter than normal respiration periods. This was 628.6 ± 677.3 s, 192.3 s for the minimum, 4064.3 s for the maximum, and 58,462.2 s for the total. For normal respiration, the time duration was 3691.8 ± 8851.8 s, 0.1 s for the minimum, 61,326.1 s for the maximum, and 1,871,720.2 s for the total. The time durations were 15.8 times the number ratio between normal and low-functional respirations. Each condition datum was sequentially listed and processed by complex-valued wavelet transform to obtain time-varied spectral series. The frequencies given by the wavelet transform were 0.01 to 5 Hz. In wavelet transform, the wavelet kernel length depends on the analytical frequency, and it was uniquely determined for each frequency. Then, the time-varied wavelet spectral series were segmented into temporal repetition with time durations of 1, 3, 5, 10, 20, 30, 60, and 90 s. These time-periodic wavelet-spectrum sequences were averaged to provide their averaged sequence. In addition, respiratory spectrum stability was statistically evaluated. The evaluation was performed for each in normal and low-functional phase by giving the average and standard deviation of the time-periodic spectral sequences. All processes were performed with software that we coded with Python software language.

### 3.2. Evaluation of the Wavelet Spectrum Variance

Correlation coefficients were given between each time-periodic wavelet-spectrum sequence and their average sequence, with respect to time duration. The time duration of data periods were each of 1, 3, 5, 10, 20, 30, 60, and 90 s. It was tested and compared between normal and low-functional respiratory conditions. In addition, it was done for each inpatient and among-patient with a 90-s time duration. The patients were 19 as introduced in Section 3.1. The correlation values were tested with Welch’s t-test. In addition, entire data collecting time-periodic data among all the patients were tested with the same statistics. Furthermore, a receiver-operating-characteristic (ROC) curve was given in the detection of low-functional respiration. It was given by increasing the threshold of normal to low-functional classification from 0.8 to 1.0 with 0.001 ticks.

## 4. Results

### 4.1. Difference Analysis between Normal and Low-Functional Respiration Periods with Wavelet Transform

**Figure 4:**
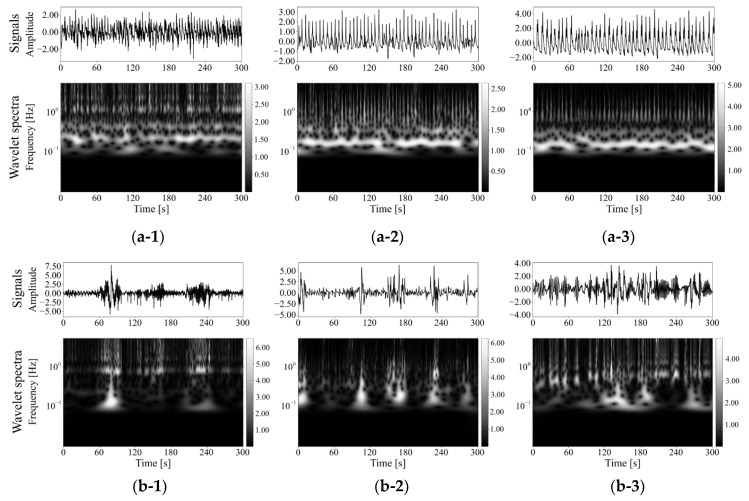
Original respiratory signals shown at the top and their wavelet spectra shown at the bottom: (**a**) Normal respiration phases and (**b**) Low function phases. The pair of each subnumber, 1, 2, or 3, means of the same patient but different respiratory conditions of (**a**,**b**).

**Figure 5:**
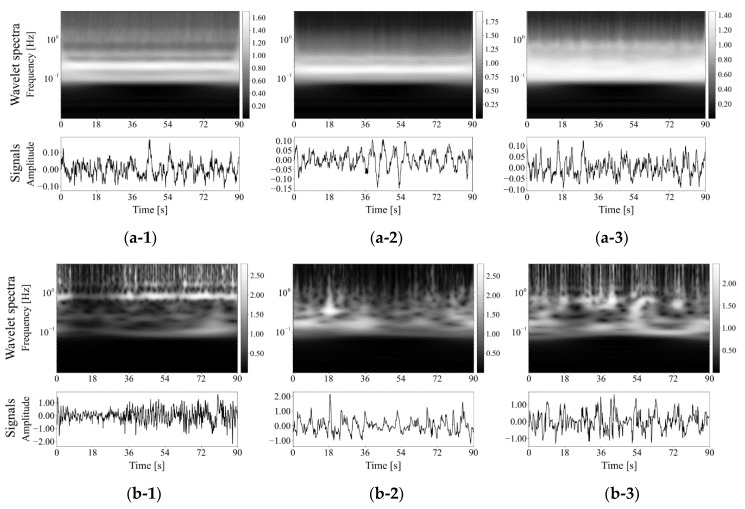
Averaged wavelet spectrum series of a 90-s period, shown at the top, and the signals reconstructed from them, shown at the bottom: (**a**) Normal respiration phases and (**b**) Low function phases. The pair of each subnumber, 1, 2, or 3, means of the same patient but different respiratory conditions of (**a**,**b**).

**Figure 6:**
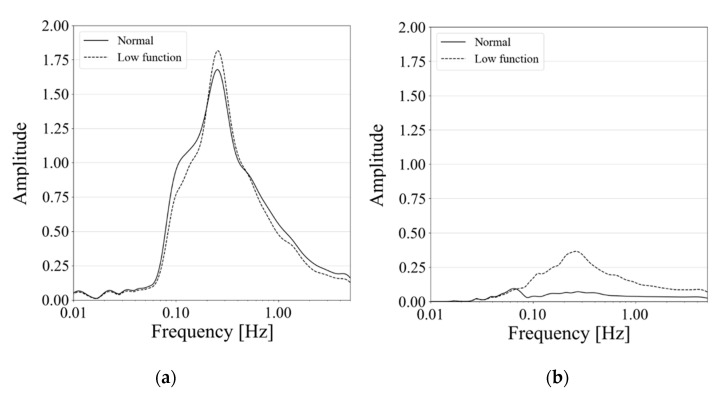
Statistics of wavelet spectra for normal and low-functional respiratory periods with respect to wavelet frequency: (**a**) Average and (**b**) Standard deviation of wavelet spectrum power.

Figure 4 shows three examples of the original signals and their wavelet spectrum of patient respiration. Wavelet spectrum in normal respiration phases did not show obvious time variation and might be flat with respect to time beside an average spectrum pattern as shown in Figure 4(a-1–a-3). The spectrum in low-functional respiration, on the other hand, was not static for time, as shown in Figure 4(b-1–b-3). They might not show any clear trend of wavelet spectrum variation for time. Figure 5 shows examples of averaged wavelet spectrum series with a 90-s duration. It means that every wavelet-spectrum time series with 90 s duration were piled and averaged at each past time in the duration. We got averaged wavelet spectrum series with each of 1, 3, 5, 10, 20, 30, and 60 s in addition to 90 s but show only the results of 90 s here because the results highlighted the difference between normal and low-functional respirations. The averaged wavelet spectrum for normal respiration looked flat and might be highly correlated with respect to time as shown in Figure 5a. For low-functional respiration, the averaged wavelet-spectrum period varied for time rather than the normal respiration as shown in Figure 5b. This might show low correspondence among them. For giving a supplement, respiratory signals reconstructed by an inverse wavelet transform are shown at the bottom line in Figure 5. No obvious trend was observed in them. Figure 6 shows the statistics of the wavelet spectrum for normal respiration periods and low functional periods. The average spectrum showed similar distributions of both normal and low-functional periods as shown in Figure 6a. The spectrum peaked around 0.25 Hz and decayed smoothly for both lower and higher frequencies. They approximately converged to zero around 1 Hz, which included 99.8% of the entire volume, for high frequencies. For low frequencies, the spectrum power might have disappeared at around 0.01 Hz, which was the 99.8% lower inclusion. The wavelet-spectrum variance was larger for low-functional respiration and around double to four times compared with normal respiration as shown in Figure 6b. It means the feasibility that the evaluation of wavelet spectrum stability could detect the timing for respiration activity to get from normal to a low functional condition. The peak of difference was at around 0.25 Hz, which corresponded with the peak of wavelet spectrum power.

### 4.2. Evaluation of Wavelet-Spectrum Variance

**Figure 7:**
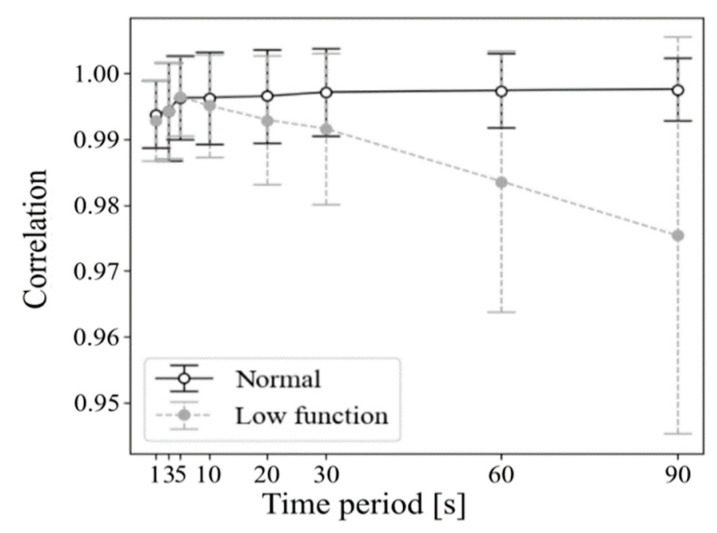
Correlation coefficient of wavelet-spectrum series with respect to time duration of each period.

**Figure 8:**
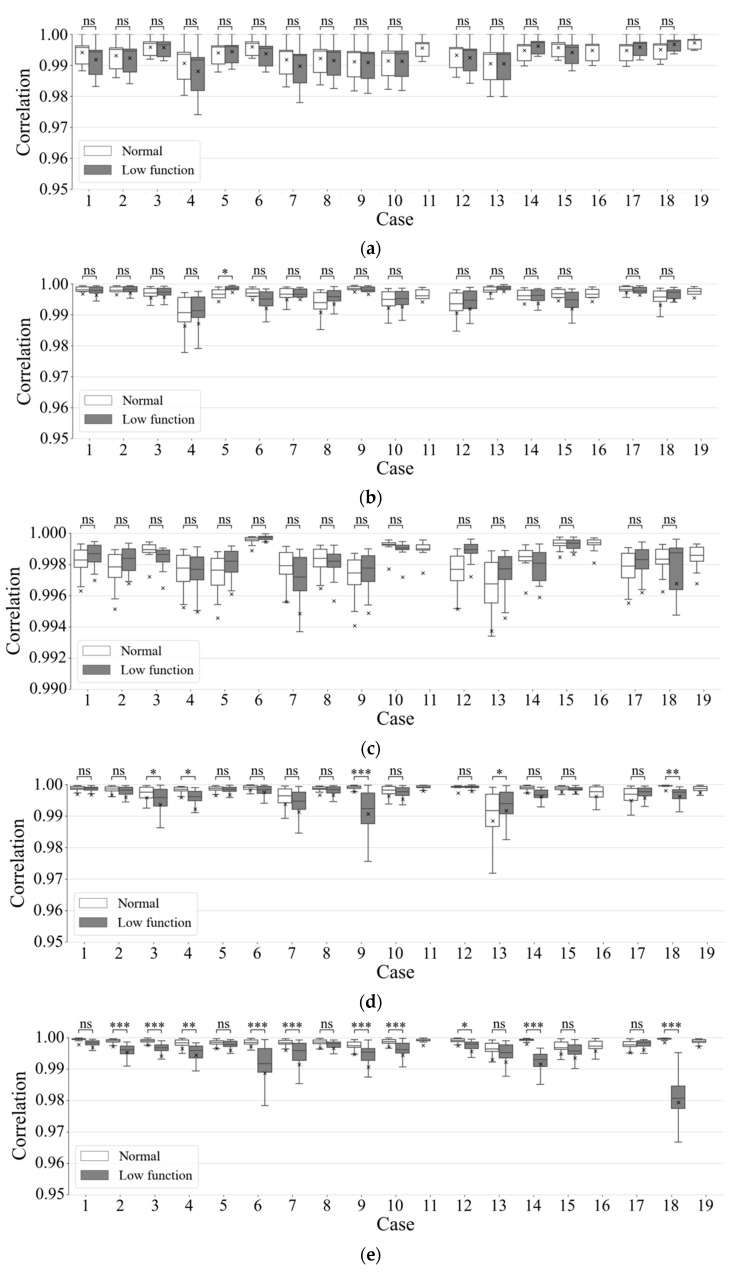

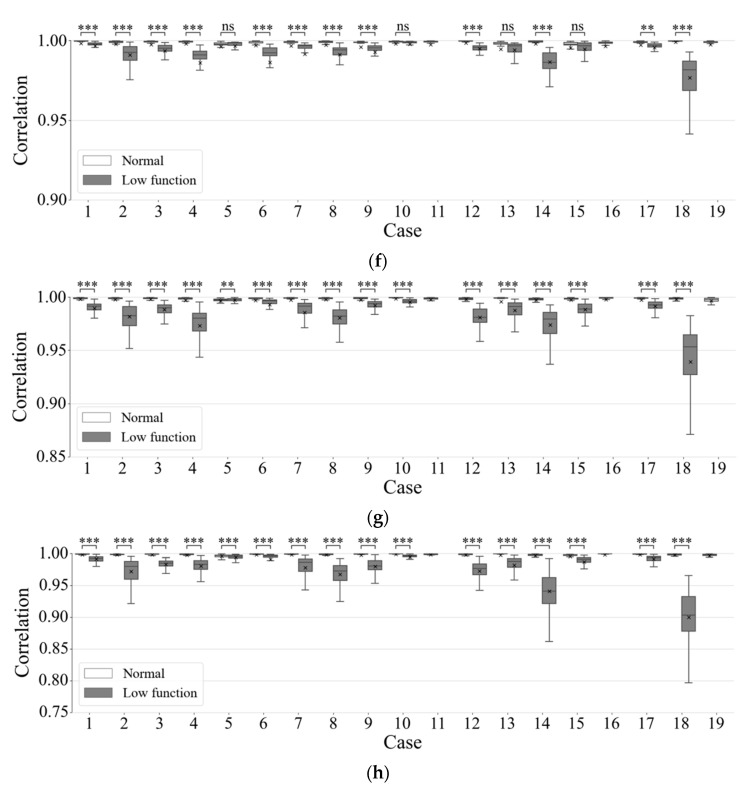
Correlation coefficients of wavelet-spectrum series with the average spectra for each patient. Time duration of wavelet-spectrum period were (**a**) 1, (**b**) 3, (**c**) 5, (**d**) 10, (**e**) 20, (**f**) 30, (**g**) 60, and (**h**) 90 s. In the figure, *, **, and *** mean statistical significances of p≤0.05, p≤0.01, and p≤0.001, respectively.

**Figure 9:**
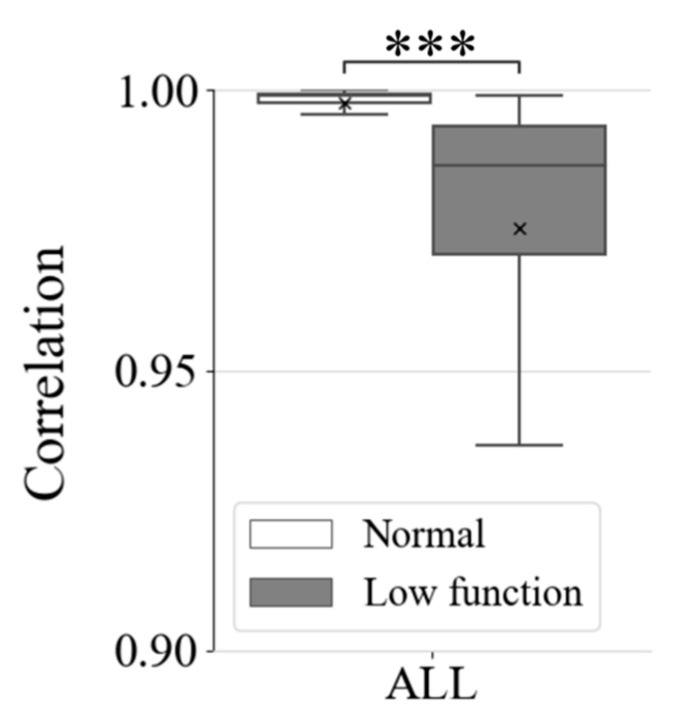
Summary of correlation coefficients of 90-s wavelet-spectrum series for all patients. In the figure, *** means statistical significance of p≤0.001.

**Figure 10:**
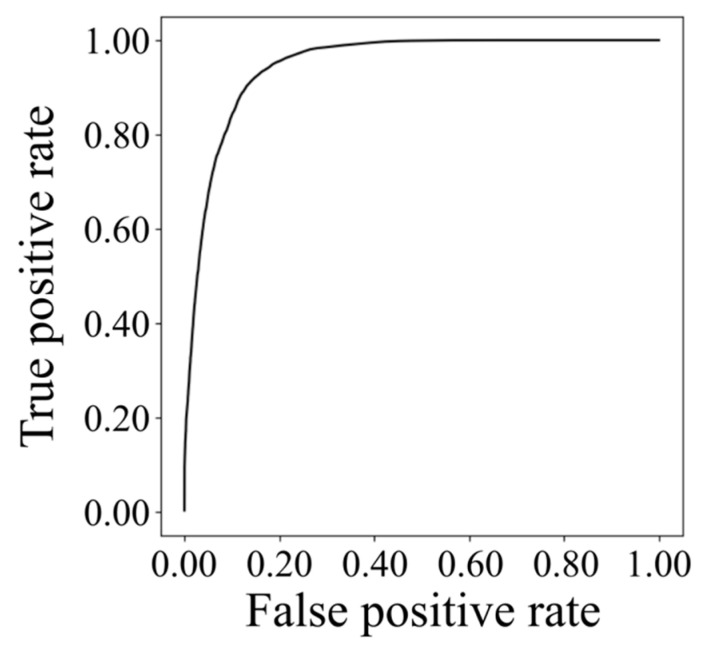
ROC curve for low-functional-respiration detection.

Figure 7 shows correlation coefficients among wavelet spectrum periods with respect to the period’s time durations, which were 1, 3, 5, 10, 20, 30, 60, or 90 s. Results showed that the correlation of normal respiration was higher than the correlation of low functional respiration was in most cases. Particularly in long time periods exceeding 10 s, a difference appeared between normal and low-functional conditions and increased as time duration became longer. Figure 8 and Figure 9 show the statistics of correlation coefficients for each normal and low-functional respiration. In Cases 11, 16, and 19, the lack of low-functional data was due to the shortness of the low-functional respiration periods. Most correlation values showed significant differences among them with Welch’s t-test in each patient’s data. The correlation values in the entire data also showed significant differences among them. Figure 10 shows the receiver operating characteristic (ROC) curve in the detection of low-functional respiration. It shows the possibility curve passing through the combinations of (0.81, 0.95) at 0.9973, (0.88, 0.88) at 0.9960, and (0.92, 0.80) at 0.9946 for (1—specificity, sensitivity) at an arbitrary threshold used in normal-to-low-functional classification.

## 5. Discussion and Conclusions

Figure 4 shows normal conditions’ stable wavelet spectra over time compared with those of low-functional respiration. The peak of the wavelet-spectrum distribution for the frequency axis appeared around 0.2 Hz, which was at a 5 s interval of respiration. The spectrum drifted to higher frequencies in the wavelet spectra of low-functional respiration. Over-time averaging highlighted such differences between the spectra of normal and low-functional conditions are shown in Figure 5. In low-functional respiration, repetitive signal patterns with a 60–80 s interval appeared weakly but not obviously. The temporal variance of spectral distribution appeared stronger in low-functional respiration than that in normal respiration.

As shown in Figure 6, the averages of spectral distributions with respect to frequency were similar for both normal and low-functional respirations. The standard deviations of spectrum distributions were small and stable for the time in normal respiration rather than in the low-functional condition. The difference between these two appeared obviously as following the time duration was getting long. This showed significant differences both in each and among all the patients as shown in Figure 8 and Figure 9.

Figure 10 shows the ROC curve in the detection of low-functional respiration. It looks like the detection for low-functional respiration would be carried out with good accuracy and reliability exceeding 0.88 for sensitivity and 0.88 for 1—specificity. The threshold can be a criterion to distinguish normal and low-functional conditions in automatic respiration monitoring. Although the criterion to determine the threshold depends on each clinical case, the result shown in Figure 10 provides useful evidence towards respiration-condition monitoring.

We proposed the processing of respiratory signals to classify the two conditions of normal and low-functional respirations. It employed a complex-valued wavelet transform, the time-periodical segmentation of its spectral data, and their normalized correlation. Although we did not apply the method to distinguish respiratory conditions in real-time, the results showed some feasibility towards the detection of low-functional respiration.

## Figures and Tables

**Table 1:** Data conditions for tests.

Patient ID	Pulmonary Disease	Operative Procedure	NOR Periods	LF Periods	Patient ID	Pulmonary Disease	Operative Procedure	NOR Periods	LF Periods
1	COPD	OT	14	4	11	Normal	OT	301	1
2	IP	OT	19	9	12	COPD	VATS	14	10
3	COPD	VATS	16	8	13	Normal	VATS	5	1
4	N/A	VATS	8	4	14	Normal	VATS	6	2
5	COPD	OT	27	14	15	IP	OT	10	6
6	COPD	VATS	14	2	16	CPFE	OT	3	8
7	COPD	VATS	12	8	17	COPD	OT	12	8
8	COPD	VATS	11	3	18	Normal	VATS	8	1
9	COPD	VATS	9	3	19	Normal	VATS	9	1
10	COPD	OT	9	7	Total	-	-	507	93

COPD: chronic obstructive pulmonary disease; IP: interstitial pneumonia; CPFE: combined pulmonary fibrosis and emphysema; OT: open thoracotomy; VATS: video-assisted thoracoscopic surgery.

## Data Availability

Data sharing is not applicable.
